# Alterations in Faecal and Serum Metabolic Profiles in Patients with Neovascular Age-Related Macular Degeneration

**DOI:** 10.3390/nu15132984

**Published:** 2023-06-30

**Authors:** Qixian Yuan, Shuai Zhu, Siqing Yue, Yuqiu Han, Guoping Peng, Lanjuan Li, Yan Sheng, Baohong Wang

**Affiliations:** 1 State Key Laboratory for Diagnosis and Treatment of Infectious Diseases, National Clinical Research Center for Infectious Diseases, Collaborative Innovation Center for Diagnosis and Treatment of Infectious Diseases, The First Affiliated Hospital, Zhejiang University School of Medicine, Hangzhou 310003, Chinaljli@zju.edu.cn (L.L.); 2 Jinan Microecological Biomedicine Shandong Laboratory, Jinan 250000, China; 3 Key Laboratory of Microbial Technology for Industrial Pollution Control of Zhejiang Province, College of Environment, Zhejiang University of Technology, Hangzhou 310032, China; 4 Research Units of Infectious Disease and Microecology, Chinese Academy of Medical Sciences, Hangzhou 310003, China; 5 Department of Neurology, The First Affiliated Hospital, Zhejiang University School of Medicine, Hangzhou 310003, China; 6 Department of Ophthalmology, The First Affiliated Hospital, Zhejiang University School of Medicine, Hangzhou 310003, China

**Keywords:** age-related macular degeneration, metabolomics, faecal, serum

## Abstract

Neovascular age-related macular degeneration (nAMD) is a common and multifactorial disease in the elderly that may lead to irreversible vision loss; yet the pathogenesis of AMD remains unclear. In this study, nontargeted metabolomics profiling using ultra-performance liquid chromatography coupled with Q-Exactive Orbitrap mass spectrometry was applied to discover the metabolic feature differences in both faeces and serum samples between Chinese nonobese subjects with and without nAMD. In faecal samples, a total of 18 metabolites were significantly altered in nAMD patients, and metabolic dysregulations were prominently involved in glycerolipid metabolism and nicotinate and nicotinamide metabolism. In serum samples, a total of 29 differential metabolites were founded, involved in caffeine metabolism, biosynthesis of unsaturated fatty acids, and purine metabolism. Two faecal metabolites (palmitoyl ethanolamide and uridine) and three serum metabolites (4-hydroxybenzoic acid, adrenic acid, and palmitic acid) were selected as potential biomarkers for nAMD. Additionally, the significant correlations among dysregulated neuroprotective, antineuroinflammatory, or fatty acid metabolites in faecal and serum and IM dysbiosis were found. This comprehensive metabolomics study of faeces and serum samples showed that alterations in IM-mediated neuroprotective metabolites may be involved in the pathophysiology of AMD, offering IM-based nutritional therapeutic targets for nAMD.

## 1. Introduction

As a multifactorial retinal disease, age-related macular degeneration (AMD) is the leading cause of irreversible vision loss among the elderly around the world, and the prevalence of AMD is predicted to increase as the population ages [1,2,3]. By 2040, the AMD prevalence is estimated to be 288 million globally, but precise effective therapeutic approaches to intervene in AMD progression remain scarce [1]. AMD can be classified into various categories based on the clinical and pathological features. Early and intermediate AMD cases typically exhibit extracellular drusen deposits of protein and lipids between the retinal pigment epithelium (RPE) and Bruch’s membrane or pigmentary changes in the macula and are mostly asymptomatic [4]. Advanced AMD can be categorized as geographic atrophic or neovascular AMD (nAMD), which results in decreased or lost central vision. Dry AMD refers to early-intermediate stage AMD and geographic atrophy, whereas nAMD, also known as wet AMD, is characterized by choroidal or retinal neurosensory layer neovascularization and may be accompanied by retinal oedema, exudation, or fibrous scarring [5,6,7]. As a widespread and debilitating eye disease, nAMD poses a serious threat to vision health. In addition to the major risk factors of genetic factors and smoking, diet has been linked to the development of AMD [8,9,10,11]. A high-fat, high-sugar diet has been shown to promote AMD [12]. In addition, dietary lipids, such as unsaturated fatty acids, may play a preventive role in the development of nAMD [13,14]. Although epidemiological studies suggest that dietary patterns play an important role in AMD risk, the mechanisms by which diet is linked to the disease remain unknown [15].

Metabolomics is a comprehensive analytical method for qualitative and quantitative detection of small molecular metabolites that can reflect complex networks of various biochemical reactions [16,17]. This high-efficiency method can be used to investigate significant biomarker signals of AMD and improve our understanding of the pathophysiology and mechanisms of this multifactorial disease [18,19]. Faecal metabolites can largely reveal the links among the host, intestinal microbiome (IM), and environmental factors (especially diet). In a laser-induced choroidal neovascularisation mouse model, researchers found a correlation between altered microbiota and faecal metabolites and reported significant changes in both the intestinal microbial composition and faecal metabolite levels [20]. Moreover, several plasma metabolomics studies have revealed that the metabolic profile changes significantly in AMD patients [21,22]. Osborn et al. pioneered the application of metabolomics for the identification of potential biomarkers of AMD [23]. Notably, the IM composition of AMD patients changed significantly in our previous study and in Zinkernagel’s study, implying that further investigation of the faecal and serum metabolic characteristics of AMD patients is required to reveal the possible pathogenesis of AMD and to provide novel therapeutic strategies [24].

In this study, nontargeted detection was performed using ultra-performance liquid chromatography coupled with Q-Exactive Orbitrap mass spectrometry (UPLC-MS) on faecal and serum samples to investigate the difference in the metabolic profiles between neovascular AMD patients and non-AMD controls. This study provides evidence to understand potential faecal and serum metabolic biomarkers in nAMD that could guide future research and clinical practice.

## 2. Materials and Methods

### 2.1. Participant Recruitment

The study was conducted according to the Declaration of Helsinki (2000) of the World Medical Association and with the approval of the ethics committee of the First Affiliated Hospital, School of Medicine, Zhejiang University, Hangzhou, China. Individuals were recruited for this research from January 2019 to May 2019 and prospectively provided written informed consent.

**Figure 1:**
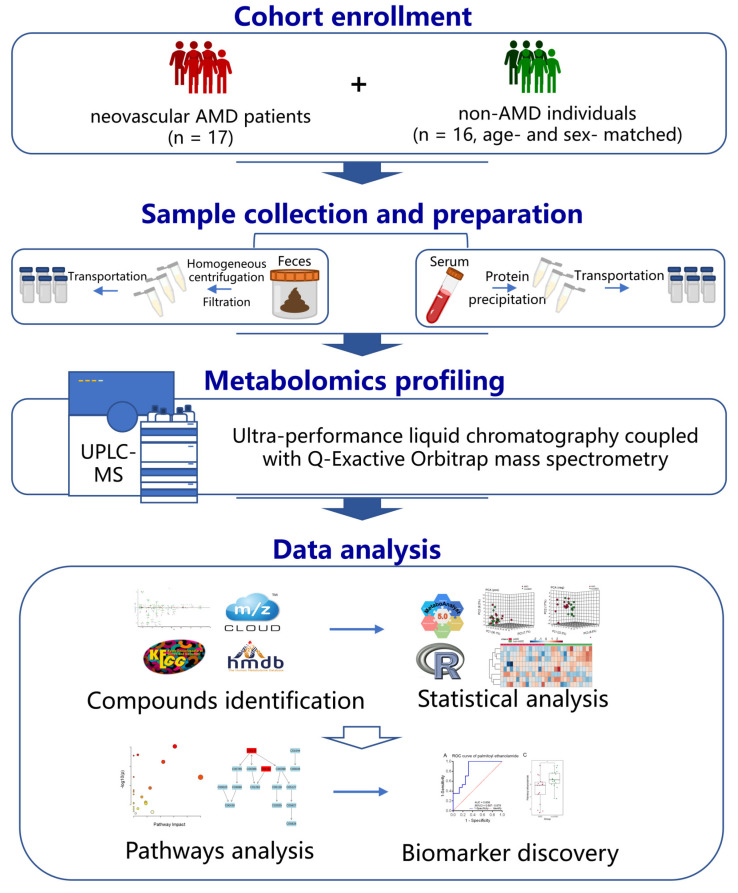
Experimental design of this study for investigating faecal and serum metabolic alterations in nAMD patients. Abbreviations: nAMD, neovascular age-related macular degeneration.

The workflow of this study is presented in Figure 1. A total of 33 nonobese and nondiabetic elderly individuals were recruited, consisting of 17 patients with nAMD from the ophthalmology department and 16 age- and sex-matched elderly individuals without nAMD as controls in the heath examination centre of our hospital. All nAMD patients were recruited without any treatment. The diagnosis of nAMD was based on the results of a comprehensive eye examination, including slit lamp microscopy, indirect fundoscopy, fundus photography, optical coherence tomography angiography, and fluorescein fundus angiography. Individuals with other causes of macular degeneration, such as trauma, uveitis, or high myopia; a history of antibiotics, bowel disease, or probiotic medication or food within 4 weeks; and a history of renal insufficiency, diabetes, blood disorders, infectious diseases, or benign or malignant tumours were excluded.

### 2.2. Clinical Data and Sample Collection

All patients with nAMD were recruited without any treatment. Demographic and clinical data were collected before sampling, including age, sex, height, weight, vascular systolic pressure, vascular diastolic pressure, fasting blood glucose, and C-reaction protein. Body mass index (BMI) was calculated. The samples were collected from the nAMD group on the reserved day for treatment (intravitreal injection of anti-vascular endothelial growth factor agents) and from the non-AMD group on the reserved day for routine health examination. The non-AMD controls did not undergo imaging testing for AMD because of the lack of any clinical symptoms.

Samples were collected from all participants on the same day in the early morning for routine clinical examination in our hospital, the residual of which was collected for metabolomics profiling. Faecal samples were obtained from all subjects, but serum samples were not obtained from 3 nAMD patients and 1 non-AMD control subject. The fasting blood samples were collected into vacuum blood collection tubes (Becton Dickinson, Franklin Lakes, NJ, USA), transported from the clinical outpatient centre to the laboratory swiftly, and then centrifuged at 3500 rpm for 10 min at 20 °C. Fresh faecal samples were collected using faecal collection containers and conveyed to the laboratory immediately at 4 °C. The supernatant and faecal samples were stored at −80 °C until metabolomics analysis (Thermo Fisher Scientific, San Diego, CA, USA).

### 2.3. Pretreatment of Serum and Faecal Samples

All samples were thawed on ice before pretreatment. For serum samples, the method for preparation was previously described [25]. Briefly, 50 μL of each serum sample was combined with 150 μL of acetonitrile (3:1 *v*/*v*). Following thorough mixing, samples were centrifuged for 10 min (12,100 rpm at 4 °C) to remove protein. Each sample supernatant (140 μL) was transferred to a sample bottle (Waters, Milford, CT, USA) with a liner for further analysis. A mixed sample of 10 μL from each sample was used as a quality control (QC) sample of faeces and serum.

For faecal samples, each sample (200 mg) was mixed with 600 μL of methanol (precooled) before being added to approximately 200 μL of ceramic beads (1 mm) (Omni International, Bedford, NH, USA) and homogenised at 8 m/s for 15 s. After thoroughly vortexing, samples were centrifuged for 10 min (10,000 rpm at 4 °C) to remove impurities. The supernatants were transferred to the new tubes for further analysis. Prior to loading, the supernatant was filtered through a filter (0.2 μm pore size) (Millipore Corp, Billerica, MA, USA) and added to a sample bottle containing the liner.

### 2.4. High-Resolution Untargeted Metabolomics Analysis of Serum Samples and Faecal Samples

The supernatants were loaded onto an Accela Open Autosampler maintained at 8 °C. Compounds in each sample were separated with a Dionex UltiMate 3000 RS ultraperformance liquid chromatography (UPLC) system on a Hypersil Gold C-18 column (2.1 × 100 mm, 1.9 µm, Thermo Fisher Scientific, Waltham, MA, USA) at 35 °C and detected on a Q-Exactive Orbitrap mass spectrometer (MS) (Thermo Fisher Scientific, San Diego, CA, USA) [25]. Briefly, the gradient mobile phase consisted of water containing 0.1% formic acid (A) and methanol containing 0.1% formic acid (B) under electrospray ionization-positive (ESI +) mode and water and methanol containing 0.1% formic acid under ionization-negative (ESI−) mode [26]. A linear elution gradient for serum samples was set as follows: 2% B during 0–0.5 min; 2–50% B during 0.5–5 min; 50–98% B during 5–10 min; 98% B during 10–15 min; and 2% B for the last 3 min. A linear elution gradient for faeces samples was set as follows: 2% B during 0–0.5 min; 2–40% B during 0.5–8 min; 40–98% B during 8–12 min; 98% B during 12–14 min; and 2% B for the last 2.5 min. A Hypersil Gold C-18 column (2.1 × 100 mm, 1.9 µm, Thermo Fisher Scientific, Waltham, MA, USA) was used in both ESI+ and ESI− mode.

Metabolite detection was performed using an Orbitrap mass spectrometer, and the acquisition mode was full MS followed by data-dependent MS2 (ddMS2) with a *m*/*z* range of 70–1050, with the standard operation procedure in our laboratory as previously described [25,26,27]. For quality control and assurance, QC samples were repeatedly tested 10 times to ensure a stable system before sampling and were run every 10 samples in the sampling process. Peak extraction and quantification of ion intensities were performed by the adaptive processing software Xcalibur 4.1 (Thermo Fisher Scientific, Waltham, MA, USA).

### 2.5. Data Analysis

Statistical analysis was performed to describe the basic characteristics of the included study population as previously described [27]. Continuous variables are summarised as the mean and standard deviation, and dichotomous or categorical variables are summarised as percentages. The differences in clinical and demographic data between the two groups were analysed in SPSS v. 21.0 (Umetrics AB, Umea, Vasterbotten, Sweden). The significance level was set to <0.05 (two-tailed).

To identify the differential metabolic features in nAMD patients, faecal and serum metabolomics data from the UPLC-MS detection system were imported into Compound Discoverer 3.3 (Thermo Fisher Scientific, Waltham, MA, USA) for matching with databases, including the MZ cloud database, Human Metabolome Database (HMDB), and Kyoto Encyclopedia of Genes and Genomes (KEGG) database. Then, the data were analysed using a comprehensive web-based platform, MetaboAnalyst 5.0 (https://www.metaboanalyst.ca/ (accessed on 18 May 2023)). Univariate analysis and multivariate analysis were used to identify the differential metabolites. Unsupervised principal component analysis (PCA) was performed to centre and scale data, while supervised partial least squares discriminant analysis (PLS-DA) was used to visualise clusters and find important features based on variable importance in projection (VIP) scores [25]. Metabolites with a *p*-value < 0.05 and VIP > 1 were considered to change significantly between the nAMD group and the control group. Pathway analysis was performed to discover the key metabolic pathways that matched the KEGG database. Furthermore, the area under the curve (AUC) of the receiver operating characteristic (ROC) curve based on the linear support vector machine (SVM) algorithm was computed to identify key metabolites with good diagnostic efficiency. Spearman’s rank test was performed for correlation analysis. Finally, Spearman’s correlation coefficients between variables were calculated using the linkET R package and dplyr R package. Correlations were plotted using the ggplot2 package.

## 3. Results

### 3.1. Clinical Characteristics of the Study Cohort Subjects with or without nAMD

This study included 33 nonobese and nondiabetic subjects (Table 1). There were 17 nAMD patients (mean age 73.18 ± 9.23 years) and 16 age- and sex-matched non-AMD controls (mean age 74.38 ± 8.816 years). There was no significant difference between the two groups, including age, sex, BMI, vascular systolic pressure, vascular diastolic pressure, fasting blood glucose, and C-reactive protein. All individuals were long-term Chinese residents of Hangzhou in China.

### 3.2. Faecal Metabolic Profiles in Subjects with or without nAMD

**Figure 2:**
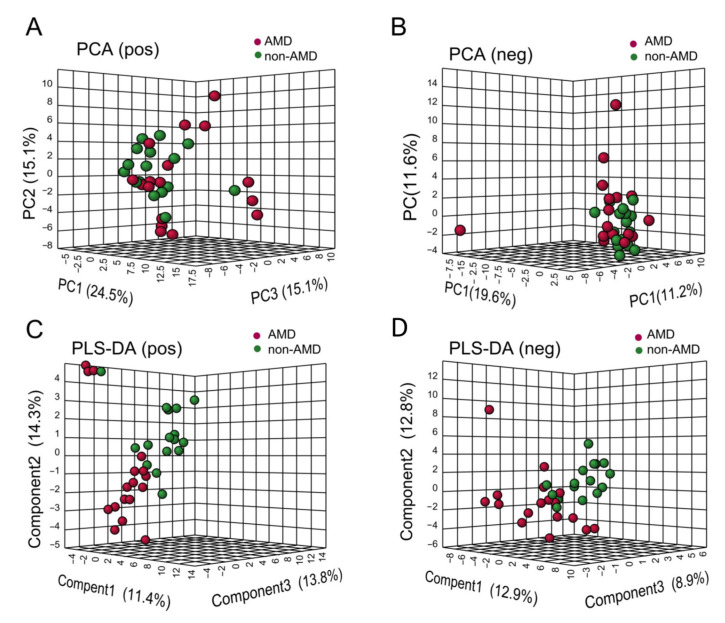
Multivariate analysis of faecal metabolomics of the nAMD and non-AMD groups detected in positive and negative ion modes. (**A**,**B**) PCA score plots were obtained from UPLC-MS data in positive and negative ion modes, respectively. (**C**,**D**) Supervised PLS-DA score plots in positive (**C**) and negative ion (**D**) modes, respectively. Abbreviations: nAMD, neovascular age-related macular degeneration; PCA, principal component analysis; PLS-DA, partial least squares discriminant analysis; UPLC-MS, ultra-performance liquid chromatography coupled with Q-Exactive Orbitrap mass spectrometry.

A total of 1922 and 1747 ion peaks were detected using Compound Discoverer 3.2 in positive ion mode and negative ion mode, respectively. After data filtering and normalisation, 100 (positive ion mode) and 89 (negative ion mode) metabolites matched with the HMDB and KEGG databases were used for univariate and supervised multivariate analysis separately to observe differences in metabolism between the two groups. As shown in Figure 2, although the faecal metabolomics PCA score plot did not display clear separation between the nAMD patient and control groups, the supervised PLS-DA analysis showed a separation between the two groups in both negative and positive ion modes (Figure 2C,D). PLS-DA models in two ion modes were not overfit according to the results of 200 permutations (Figure S1).

**Figure 3:**
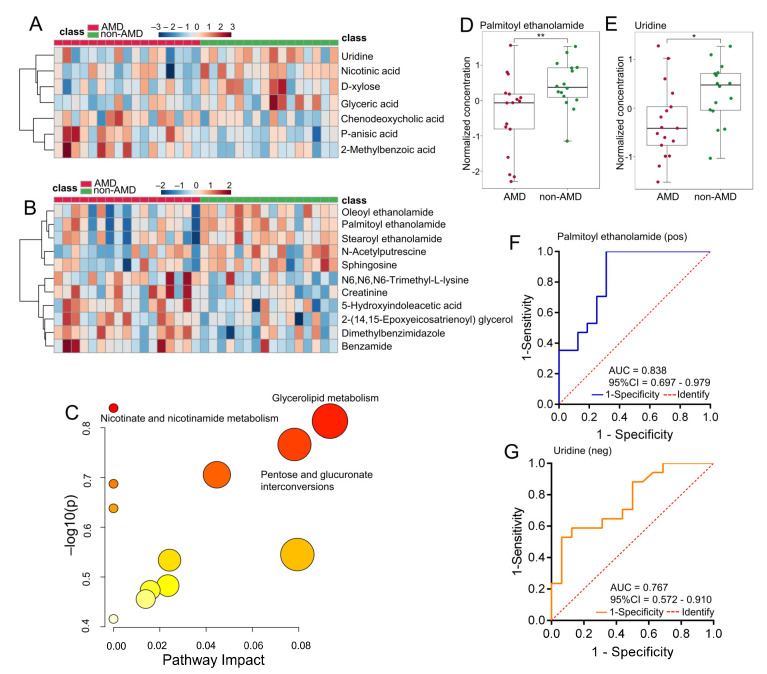
Differential faecal metabolic features of nAMD patients. (**A**,**B**) Hierarchical clustering heatmaps of faecal metabolites with significant differences (*p*-value < 0.05 and VIP score >1) between nAMD patients and non-AMD controls detected in positive and negative ion modes, respectively. The red or blue colour in each cell represents whether the levels of each metabolite were high or low, respectively. (**C**) Changes in faecal metabolomic pathways, mainly including glycerolipid metabolism, nicotinate and nicotinamide metabolism, and pentose and glucuronate interconversions. The bubbles represent the enriched pathways of metabolites and the color represents metabolites intensity from low (light yellow) to high (deep red). (**D**,**E**) The relative concentrations of palmitoyl ethanolamide (positive ion mode) and uridine (negative ion mode) in the nAMD group and non-AMD group. The color of dots represents different group: red, AMD; green, non-AMD. *, *p*-value < 0.05; **, *p*-value < 0.01. (**F**,**G**) ROC curve of two selected faecal biomarkers with the best diagnostic capacity. Abbreviations: nAMD, neovascular age-related macular degeneration; VIP, variable importance in projection; ROC, receiver operating characteristic; AUC, area under the curve; CI, confidence interval.

In positive ion mode and negative ion mode, 7 and 11 significantly changed metabolites with *p*-value < 0.05 and VIP > 1 were selected, respectively, and the relative levels of these metabolites are shown in Figure 3A,B. As shown in Figure 3C, the main affected pathways of faecal metabolites were nicotinate and nicotinamide metabolism, glycerolipid metabolism, and pentose and glucuronate interconversions.

To identify the different faecal metabolites of nAMD, we performed a biomarker analysis to explore the diagnostic capacity of faecal metabolic features. The AUCs and related 95% CIs of 18 differential faecal metabolites based on ROC analysis are shown in Supplementary Table S1. As shown in Figure 3F,G, palmitoyl ethanolamide (PEA) was detected in positive ion mode (AUC, 0.838; 95% CI, 0.697–0.979) and uridine was detected in negative ion mode (AUC, 0.767; 95% CI, 0.572–0.910) with better accuracy than other metabolites.

### 3.3. Serum Metabolomic Profiles in Subjects with or without nAMD

**Figure 4:**
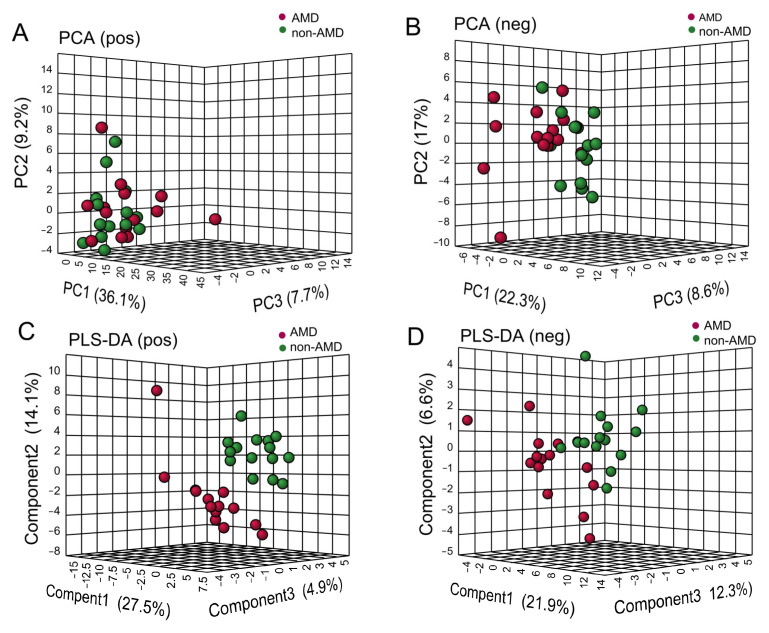
Multivariate analysis of serum metabolomics of the nAMD and non-AMD groups detected in positive and negative ion modes. (**A**,**B**) PCA score plots were obtained from UPLC-MS data in positive and negative ion modes, respectively. (**C**,**D**) Supervised PLS-DA score plots in positive and negative ion modes, respectively. Abbreviations: nAMD, neovascular age-related macular degeneration; PCA, principal component analysis; PLS-DA, partial least squares discriminant analysis; UPLC-MS, ultra-performance liquid chromatography coupled with Q-Exactive Orbitrap mass spectrometry.

A similar analytical setup was used for serum metabolite exploration. A total of 6575 and 2995 ion peaks were detected using Compound Discoverer 3.2 in positive ion mode and negative ion mode, respectively, and 95 (positive ion mode) and 59 (negative ion mode) metabolites matched with the HMDB and KEGG databases were used to perform statistical analysis to find metabolites that were significantly changed after data preparation. The PCA and PLS-DA score plots are shown in Figure 4, revealing that the separation of the nAMD and non-AMD groups was distinct in both positive and negative ion modes. The permutation test of the two ion modes showed that the models were not overfitted (Supplementary Figure S2).

**Figure 5:**
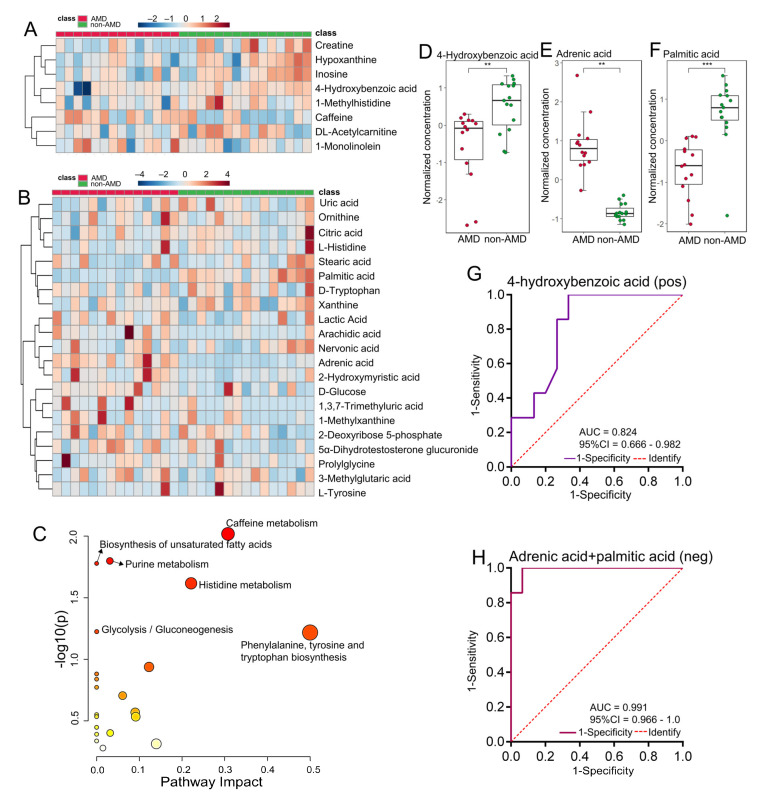
Differential serum metabolic features of nAMD patients. (**A**,**B**) Hierarchical clustering heatmaps of serum metabolites with significant differences (*p*-value < 0.05 and VIP score >1) between nAMD patients and non-AMD controls detected in positive and negative ion modes, respectively. The red or blue colour in each cell represents whether the level of each metabolite was high or low, respectively. (**C**) Changed serum metabolomic pathways, mainly including glycerolipid metabolism, nicotinate and nicotinamide metabolism, and pentose and glucuronate interconversions. The bubbles represent the enriched pathways of metabolites and the color represents metabolites intensity from low (light yellow) to high (deep red). (**D**–**F**) The relative levels of 4-hydroxybenzoic acid (positive ion mode) and adrenic acid and palmitic acid (negative ion mode) in the nAMD group and non-AMD group. The color of dots represents different group: red, AMD; green, non-AMD. **, *p*-value < 0.01; ***, *p*-value < 0.001. (**G**,**H**) ROC curve of selected serum biomarkers with the best diagnostic capacity. Abbreviations: nAMD, neovascular age-related macular degeneration; VIP, variable importance in projection; ROC, receiver operating characteristic; AUC, area under the curve; CI, confidence interval.

Differential metabolites were identified according to both VIP score > 1 in PLS-DA and *p*-value < 0.05. The levels of these metabolites obtained in both positive and negative ion modes are shown in Figure 5A,B. In the enriched metabolic pathway analysis, the top three perturbed metabolic pathways were mainly related to caffeine metabolism, biosynthesis of unsaturated fatty acids, and purine metabolism (Figure 5C). ROC relative analysis was performed based on differential metabolites, and the results are shown in Table S2. Figure 5D–F show the alterations in three metabolites with the best AUC values in serum samples of nAMD patients, and Figure 5G–H show their discriminatory abilities. We demonstrated that 4-hydroxybenzoic acid (4-HBA) (AUC, 0.824; 95% CI, 0.666–0.982) in positive ion mode and a model including adrenic acid (AdA) and palmitic acid (PA) (AUC, 0.991; 95% CI, 0.966–1.0) in negative ion mode performed the best.

### 3.4. Correlation Analysis among Altered Faecal Metabolites, Serum Metabolites, and IM of nAMD

**Figure 6:**
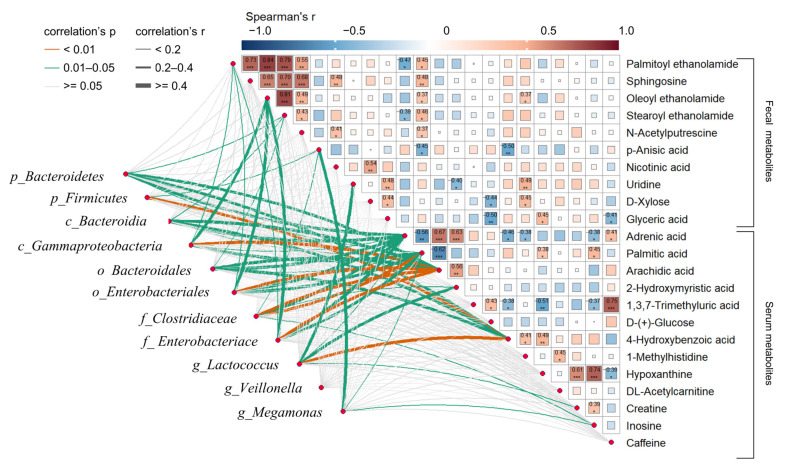
Correlations between altered metabolites (AUC > 0.7) and the intestinal microbiota of nAMD patients and non-AMD controls. Spearman’s rank correlation between faecal and serum metabolites and the intestinal microbiota. The line width corresponds to the Spearman correlation, and line colour denotes the statistical significance based on 999 permutations. Pairwise comparisons of metabolites are also shown, with a colour gradient denoting Spearman’s correlation coefficient. The colour and thickness of the lines represent the strength of the correlation between the metabolites and the differential microbiome. The thicker the line is, the stronger the correlation. The orange colour represents strong significance, *p*-value < 0.01. The correlation heatmap in the upper right corner indicates the correlation relationship between different important metabolites. Numbers indicate the correlation r value and asterisks indicate statistical significance (*** *p*-value < 0.001; ** *p*-value < 0.01; and * *p*-value < 0.05).

To explore the functional linkages between differential IM and metabolites of nAMD, Spearman’s correlation coefficient analysis was performed among different faecal metabolites, serum metabolites, and the IM of nAMD, which was identified in our previous study, as shown in Figure 6. Of note, we found that there were significant linkages among a total of 23 metabolites (AUC > 0.7) and different IM in nAMD. For example, significant positive correlations were found among faecal metabolites themselves, such as PEA, oleoyl ethanolamide (OEA), stearoyl ethanolamide (SEA), sphingosine, and n-acetylputrescine. Moreover, significant correlations were also found between faecal and serum metabolites. Interestingly, the serum metabolites adrenic acid, palmitic acid, and arachidic acid had strong linkages with the altered microbes in nAMD. Among them, serum 4-HA strongly correlated with multiple differential IM of nAMD. In addition, the faecal metabolites PEA and OEA had significant correlations with the disturbed IM of nAMD, especially opportunistic pathogens at all levels (*c_Gammaproteobacteria*, *o_Enterobacteriales*, and *f_Enterobacteriace*). The findings demonstrated the close correlation between disturbed microbiota composition and altered host metabolism in nAMD.

## 4. Discussion

In this study, we focused on characterising the altered faecal and serum metabolic profiles in nonobese and nondiabetic nAMD patients and their linkages with the different IM of nAMD. The findings demonstrated that the altered human metabolism of nAMD in both faeces and serum, together with the different IM, was significantly linked. These findings indicated that these metabolic disturbances as well as the altered microbiota might play an important role in promoting nAMD development.

Here, the main finding in faecal metabolomics profiles of our nAMD patients was the decreased level of neuroprotective metabolites, such as N-acylethanolamines (NAEs), including PEA, OEA, and SEA. NAEs could be involved in maintaining the homeostasis of the intestinal environment and host metabolism [27], regulate the IM composition and gut barrier function [28], and be effective at activating neuroprotective mechanisms to suppress inflammatory neurodegenerative disease [29]. More importantly, NAEs and their receptors were found in the retina [30]. Consistent with the previous findings of the altered microbiome in nAMD patients [31], our findings firstly reported lowered NAEs in the faecal metabolome of nAMD patients, which might be caused by diet and IM and closely related to the disease process [32,33,34]. Among NAEs, PEA is an endogenous PA amide with anti-inflammatory activity, neuroprotective effects, and retinoprotectant capacity [35,36,37]. PEA can protect cells from damage and can promote immune system balance [38]. In this study, we found that PEA together with uridine could be used to distinguish nAMD from non-AMD subjects. In line with our results, PEA supplementation reduced retinal neovascularisation and fibrotic changes in a mouse model of oxygen-induced retinopathy [39]. In agreement with our human nAMD findings, a laser-induced choroidal neovascularisation mouse model study reported that the gut microbiome of AMD mice exhibited significant alterations, leading to significant changes in metabolomic profiles, such as uridine [20]. Uridine, a necessary pyrimidine nucleotide for RNA synthesis, has been widely used in reducing cytotoxicity and improving neurophysiological functions [40]. Thus, the decreased level of these reduced neuroprotective NAEs and uridine could be novel targets for nAMD treatment.

Furthermore, the serum metabolomics profiles of our nAMD patients presented the significantly decreased neuroprotective 4-HBA and lipid PA and enriched unsaturated fatty acid AdA, which were identified as the potential serum biomarkers for nAMD in the study. 4-HBA in serum samples could be derived from the catechin metabolism of green tea, which is involved in ubiquinone biosynthesis in humans and could inhibit oxidative stress, thus protecting neuronal cells in neurodegeneration [41]. PA is a major fatty acid existing in human blood and retina and interferes with multiple normal biological functions, including protein palmitoylation and PEA biosynthesis [42,43], which, in turn, influence the oligodendrocyte differentiation and are involved in age-related neurodegenerative disorders [44]. Our results of lower serum PA and faecal PEA levels in the nAMD group highly indicated that diet-nutrition-adjustment treatment could improve the neuroprotective metabolites in nAMD patients and provide remission of the AMD disease process.

Notably, the significant functional linkages between differential IM and metabolites of faeces and serum in our nAMD were found**.** The results of our study evidenced a role of the IM in the mechanism and metabolic pathways of the pathogenesis of nAMD, which provided a novel therapeutic target for nAMD. In consistence with our findings, Andriessen et al. reported a novel link between homeostasis of gut microbiota and ocular angiogenesis, in which a prolonged high-fat diet and obesity could cause dysbiosis, lead to increased intestinal permeability and inflammation characterized by chronic low-grade inflammation, and ultimately affect the development of neovascular lesions associated with AMD [45]. In addition, a unique transcriptomic profile had been identified in germ-free mouse retinas, demonstrating the possible existence of a unique gut microbiome–RPE/choroid axis and that the absence of the microbiome was associated with decreased neovascular lesion formation and associated inflammation [46]. In agreement with our findings in nAMD patients, Li and colleagues identified an altered faecal microbiome and metabolome in a CNV mouse model that might be associated with the pathogenesis of nAMD [20]. The emerging evidence had delineated a potential role of intestinal microbiota in nAMD, but the mechanism by which the microbiome modulates retinal functions remains unclear. Our results of the covariation among the IM, faecal metabolome, and serum metabolome in nAMD indicated that metabolites can be the mediator of the interaction among the diet, IM, and host, reflecting the systemic metabolic and dietary status of nAMD. Our results showed that the significant decreased neuroprotective and antineuroinflammatory metabolites, such as PEA, and 4-HBA of serum in nAMD patients had significant correlations with the serum metabolites and disturbed IM, such as Bacteroidetes and Gammaproteobacteria. These findings supported a contributory role of the IM in neovascular AMD pathogenesis by promoting low-grade inflammation via unbalancing the neuroprotective metabolites. Diet can affect the structure and function of the gut microbiota, which, in turn, may affect metabolite levels in the blood and tissues [47]. Thus, these results indicated that the IM-dysbiosis-mediated deleterious metabolic disturbance in nAMD patients could play a crucial role in nAMD.

Moreover, the disturbed faecal metabolic pathways in nAMD patients included nicotinate and nicotinamide metabolism and glycerolipid metabolism in this study. In agreement with our findings, alterations in the nicotinate and nicotinamide metabolism pathway have been found in serum metabolites of nAMD patients [48]. Nicotinic acid, also known as vitamin B3, can be converted into the coenzyme nicotinamide adenine dinucleotide and nicotinamide adenine dinucleotide phosphate for auxiliary cellular redox reactions and exhibits inflammatory activity [49]. The decreased nicotinic acid levels in nAMD faeces were similar to the reported levels in the faeces of diabetic retinopathy patients [50]. Supplementation with nicotinic acid can help vasodilation [51] and may be useful for treating nAMD and other ocular diseases, which suggests its potential clinical efficiency in nAMD.

Importantly, the dysregulation of serum fatty acid biosynthesis was found in our nAMD patients, including enriched AdA and depleted PA and stearic acid (SA). AdA is an omega-6 polyunsaturated fatty acid that is derived from the arachidonic acid chain and plays an important role in protecting human retinal function and visual development [52,53,54]. However, high levels of AdA may enhance inflammation, increasing the risk of a variety of diseases [55]. PA and SA are the two main lipids in the retinas of healthy elderly individuals [56,57]. PA is derived from dietary intake, transformations of other fatty acids, or endogenous synthesis of carbohydrates and amino acids and has been linked to neurodegenerative diseases [58]. PA can provide fuel for mitochondrial capacity and is involved in lipid and energy metabolism in the retina, while SA can activate mitochondrial function [59]. The association between decreased PA and SA levels and nAMD is still unknown and needs to be explored in future investigation.

Purine metabolism and amino acid metabolism were disturbed in serum samples of our nAMD patients. Consistent with our findings, Inês Laíns et al. reported a metabolomics study of two cohorts from the United States and Spain that revealed significant enrichment in purine and amino acid metabolism [60]. Purinergic signalling disorder may induce oxidative stress and the death of photoreceptor and RPE cells via the overactive receptor P2X, which can reduce oxidative-stress-induced accumulation [61], and contribute to macular degeneration and other retinal diseases [62], indicating potential therapeutic targets for AMD. In addition, several metabolomics studies have reported derangements of amino acid metabolism in patients with retinal disorders in plasma and even local retinal samples of humans and rats, suggesting an association between systemic metabolism and ocular metabolism [63,64,65]. Consistent with a previous report [66], decreased levels of the glycolysis-related metabolite citric acid were found in our nAMD patients. These findings indicated that significantly disturbed energy metabolism was associated with nAMD patients, which might contribute to the pathophysiology of nAMD.

Interestingly, we first found that caffeine metabolism was disturbed in nAMD patients. Caffeine at low molecular quantities in human peripheral fluids, derived from food or beverages, is primarily metabolised in the liver and can affect neurons [67]. Whereas caffeine has antioxidative efficiency, oral intake of caffeine at a dose of 300 mg after one hour can lead to ocular vasoconstriction and an increase in the resistive index of the central retina in healthy people [68]. In addition, four heavy coffee drinkers developed acute macular neuroretinopathy expansion in the clinical spectrum [69]. Little is known about the clear effects of caffeine on nAMD, and our findings provide a clue into the changing trends of caffeine in nAMD patients, which need further investigation.

There were some limitations of our study. First, the sample size was relatively small and a study with a larger sample size is needed to validate our findings. However, the nonobese and nondiabetic nAMD subjects were recruited in this study to deplete the confounding factors and decipher the potential role of the IM in nAMD, since obesity and diabetes remain high risk factors for nAMD [70] and represent the major determinants of compositional changes in microbial communities. Second, we focused on the changes in the faecal and serum metabolic profiles of nAMD patients in comparison with non-AMD control subjects in the study. nAMD patients in multiple stages should be involved to improve the understanding of the metabolic features in different courses of AMD and to find early biomarkers for targeted clinical treatments. Last, the diet pattern should be considered to confirm the potential function of diet in nAMD progression and to explore the relationship between IM alteration and systemic metabolism. Despite the limitations, our study provides novel findings on the altered faecal and serum metabolic pathways in nAMD patients and contributes to shedding light on the role of the IM in the pathogenesis of nAMD.

## 5. Conclusions

nAMD may lead to irreversible vision loss in elderly individuals; yet the exact pathophysiology of nAMD remains unknown and an efficient treatment is lacking. To our knowledge, this is the first comprehensive metabolomics study to evaluate both the faecal and serum metabolic alterations in nAMD patients in comparison with those in non-AMD subjects using an untargeted metabolomics method. This study demonstrated that metabolic dysregulation was associated with IM dysbiosis and may be involved in the development of nAMD pathology. These results indicated that the deleterious metabolic disturbance could be modified by therapeutically targeting the microbiota for nAMD treatment.

## Figures and Tables

**Table 1:** Demographic and clinical parameters of subjects of the study cohorts.

Characteristics	nAMD	Non-AMD	*p*-Value	FDR
	(*n* = 17)	(*n* =16)		
Age (years, mean ± SD)	73.2 ± 9.2	74.4 ± 8.8	0.652	0.843
Gender (% Female)	4 (23.5%)	9 (56.3%)	0.052	0.208
BMI (kg/m^2^, mean ± SD)	23.6 ± 2.2	23.5 ± 3.8	0.843	0.843
Vascular systolic pressure (mmHg, mean ± SD)	132.2 ± 18.7	124.7 ± 17.8	0.2	0.428
Vascular diastolic pressure (mmHg, mean ± SD)	73.3 ± 12.9	75.6 ± 16.4	0.843	0.843
Fasting blood glucose (mmol/L, mean ± SD)	6.3 ± 0.9	6.0 ± 0.6	0.214	0.428
C-reactive protein (mg/L, mean ± SD, CRP)	1.5 ± 1.3	1.6 ± 2.1	0.461	0.737

Note: nAMD, neovascular age-related macular degeneration; BMI, body mass index; FDR, false discovery rate.

## Data Availability

Not applicable.

## Supplementary Materials

The following supporting information can be downloaded at: https://www.mdpi.com/article/10.3390/nu15132984/s1, Figure S1: The 200 times permutation test with a 200 of PLS-DA model of faecal metabolites; Figure S2: The 200 times permutation test of PLS-DA model of serum metabolites; Table S1: ROC analysis of significant faecal metabolites detected in cationic and anionic mode; Table S2: ROC analysis of significant serum metabolites detected in positive and negative ion mode.
