# Supplementary material for: Alterations in Faecal and Serum Metabolic Profiles in Patients with Neovascular Age-Related Macular Degeneration
Source: Nutrients. 2023 Jun 30;15(13):2984. doi: 10.3390/nu15132984 (PMC10346323; doi:10.3390/nu15132984)

Supporting Information

Supplementary Tables

**Supplementary Table S1**. ROC analysis on the differential fecal metabolites of AMD detected in positive and negative ion mode.

**Supplementary Table S2**. ROC analysis on the differential serum metabolites detected in positive and negative ion mode.

**Supplementary Figures**

**Supplementary Figure S1**. Permutation test of the PLS-DA model in the (A) positive and (B) negative ion modes based on the fecal metabolomics data of the study cohort. The R2Y value represents the goodness of fit of the model. The Q2 value represents the predictability of the models. All R2Y and Q2 values to the left were lower than the original points to the right of the blue regression line of the Q2 points, which intersects the vertical axis (on the left) at or below zero, showing that the PLS-DA model was valid. Permutation test plot for the PLS-DA model (number of permutations, 200; intercepts R2=0.0, 0.708, Q2=0.0, -0.209 in ESI+; R2=0.0, 0.677, Q2=0.0, -0.15 in ESI−). ESI, electrospray ionization; PLS-DA, partial least squares-discriminant analysis.

**Supplementary Figure S2**. Permutation test of the PLS-DA model in the (A) positive and (B) negative ion modes based on the serum metabolomics data of the study cohort. The R2Y value represents the goodness of fit of the model. The Q2 value represents the predictability of the models. All R2Y and Q2 values to the left were lower than the original points to the right of the blue regression line of the Q2 points, which intersects the vertical axis (on the left) at or below zero, showing that the PLS-DA model was valid. Permutation test plot for the PLS-DA model (number of permutations, 200; intercepts R2=0.0, 0.64, Q2=0.0, -0.159 in ESI+; R2=0.0, 0.829, Q2=0.0, -0.162 in ESI−). ESI, electrospray ionization; PLS-DA, partial least squares-discriminant analysis.

**Supplementary Tables**

**Supplementary Table S1**. ROC analysis on the differential fecal metabolites of AMD detected in positive and negative ion mode calculated.

| Ion mode | Metabolites | AUC | SE | p-value | 95% CI |
|---|---|---|---|---|---|
| Positive ion mode | Palmitoyl ethanolamide | 0.838 | 0.072 | 0.001 | 0.697-0.979 |
| | Stearoyl ethanolamide | 0.79 | 0.078 | 0.004 | 0.638-0.943 |
| | Sphingosine | 0.765 | 0.09 | 0.009 | 0.588-0.941 |
| | Oleoyl ethanolamide | 0.746 | 0.085 | 0.016 | 0.579-0.913 |
| | N-Acetylputrescine | 0.735 | 0.088 | 0.021 | 0.564-0.907 |
| | 2-(14,15-Epoxyeicosatrienoyl) glycerol | 0.482 | 0.103 | 0.857 | 0.28-0.683 |
| | 5-Hydroxyindoleacetic acid | 0.415 | 0.102 | 0.407 | 0.216-0.615 |
| | N6,N6,N6-Trimethyl-L-lysine | 0.375 | 0.101 | 0.221 | 0.177-0.573 |
| | Creatinine | 0.467 | 0.104 | 0.746 | 0.264-0.67 |
| | Benzamide | 0.463 | 0.103 | 0.719 | 0.262-0.664 |
| | Dimethylbenzimidazole | 0.456 | 0.103 | 0.666 | 0.255-0.657 |
| Negative ion mode | Uridine | 0.768 | 0.082 | 0.009 | 0.608-0.929 |
| | Nicotinic acid | 0.761 | 0.086 | 0.011 | 0.593-0.929 |
| | p-Anisic acid | 0.741 | 0.086 | 0.019 | 0.572-0.91 |
| | D-Xylose | 0.746 | 0.085 | 0.016 | 0.579-0.914 |
| | Glyceric acid | 0.746 | 0.088 | 0.016 | 0.574-0.918 |
| | 2-Methylbenzoic acid | 0.305 | 0.091 | 0.056 | 0.126-0.484 |
| | Chenodeoxycholic Acid | 0.316 | 0.096 | 0.072 | 0.129-0.503 |

Notes: AMD, age-related macular degeneration; ROC, receiver operating characteristic; AUC, area under the curve; CI, confidence interval;

**Supplementary Table S2**. ROC on the differential serum metabolites detected in positive and negative ion mode.

| Ion mode | Metabolites | AUC | SE | p-value | 95% CI |
|---|---|---|---|---|---|
| Positive ion mode | 4-Hydroxybenzoic acid | 0.819 | 0.082 | 0.003 | 0.658 - 0.98 |
| | 1-Methylhistidine | 0.762 | 0.092 | 0.016 | 0.581 - 0.942 |
| | Creatine | 0.743 | 0.096 | 0.026 | 0.555 – 0.93 |
| | Inosine | 0.724 | 0.096 | 0.04 | 0.536 – 0.911 |
| | Caffeine | 0.724 | 0.095 | 0.04 | 0.538-0.910 |
| | Hypoxanthine | 0.719 | 0.099 | 0.045 | 0.526 – 0.912 |
| | DL-Acetylcarnitine | 0.71 | 0.11 | 0.055 | 0.493 – 0.926 |
| | 1-Monolinolein | 0.319 | 0.109 | 0.097 | 0.106 – 0.532 |
| Negative ion mode | Adrenic acid | 0.981 | 0.02 | <0.0001 | 0.943-1.0 |
| | Palmitic acid | 0.938 | 0.06 | <0.0001 | 0.82-1.0 |
| | Arachidic acid | 0.838 | 0.079 | 0.002 | 0.683-0.993 |
| | 2-Hydroxymyristic acid | 0.757 | 0.095 | 0.018 | 0.571- 0.943 |
| | 1,3,7-Trimethyluric acid | 0.748 | 0.096 | 0.023 | 0.56-0.936 |
| | D-(+)-Glucose | 0.738 | 0.101 | 0.029 | 0.541-0.935 |
| | Lactic Acid | 0.69 | 0.103 | 0.081 | 0.489-0.892 |
| | 5alpha-Dihydrotestosterone glucuronide | 0.686 | 0.103 | 0.089 | 0.483-0.888 |
| | 1-Methylxanthine | 0.667 | 0.104 | 0.127 | 0.464-0.87 |
| | 3-Methylglutaric acid | 0.605 | 0.107 | 0.337 | 0.396-0.814 |
| | 2-Deoxyribose 5-phosphate | 0.581 | 0.109 | 0.458 | 0.368-0.794 |
| | Prolylglycine | 0.581 | 0.109 | 0.458 | 0.368-0.792 |
| | Ornithine | 0.51 | 0.113 | 0.93 | 0.287-0.7732 |
| | Citric acid | 0.481 | 0.113 | 0.861 | 0.259-0.703 |
| | Nervonic acid | 0.486 | 0.112 | 0.896 | 0.266-0.705 |
| | D-(+)-Tryptophan | 0.419 | 0.109 | 0.458 | 0.205-0.633 |
| | Uric acid | 0.39 | 0.107 | 0.315 | 0.181-0.6 |
| | Xanthine | 0.352 | 0.112 | 0.176 | 0.133-0.572 |
| | L-Histidine | 0.343 | 0.107 | 0.15 | 0.134-0.552 |
| | L-Tyrosine | 0.319 | 0.102 | 0.097 | 0.120.518 |

Notes: AMD, age-related macular degeneration; ROC, receiver operating characteristic; SVM, support vector machine; AUC, area under the curve; CI, confidence interval.

**Supplementary Figures**

**Supplementary Figure S1**. Permutation test of the PLS-DA model in the (A) positive and (B) negative ion modes based on the fecal metabolomics data of the study cohort. The R2Y value represents the goodness of fit of the model. The Q2 value represents the predictability of the models. All R2Y and Q2 values to the left were lower than the original points to the right of the blue regression line of the Q2 points, which intersects the vertical axis (on the left) at or below zero, showing that the PLS-DA model was valid. Permutation test plot for the PLS-DA model (number of permutations, 200; intercepts R2=0.0, 0.708, Q2=0.0, -0.209 in ESI+; R2=0.0, 0.677, Q2=0.0, -0.15 in ESI−). ESI, electrospray ionization; PLS-DA, partial least squares-discriminant analysis.

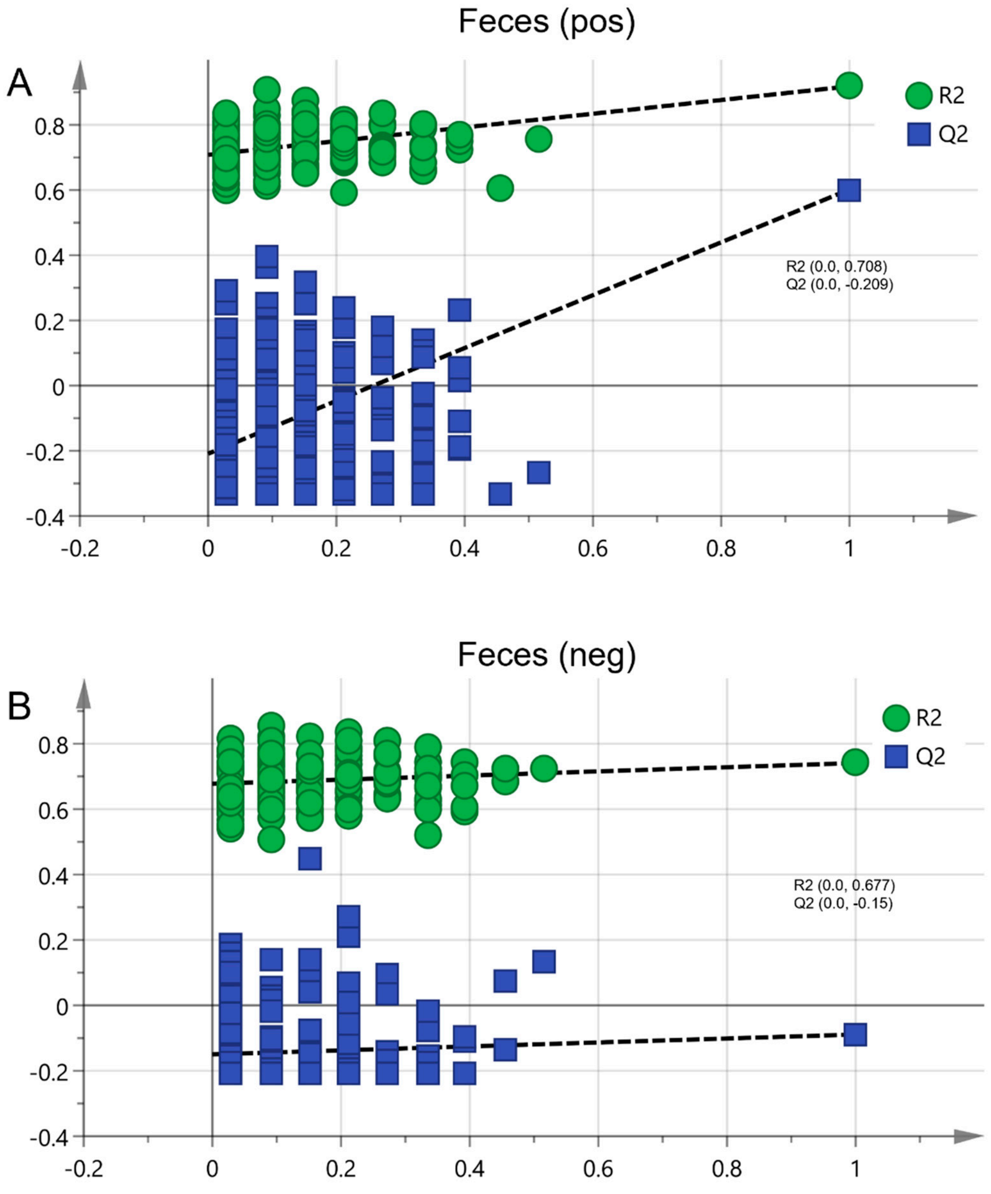

**Supplementary Figure S2**. Permutation test of the PLS-DA model in the (A) positive and (B) negative ion modes based on the serum metabolomics data of the study cohort. The R2Y value represents the goodness of fit of the model. The Q2 value represents the predictability of the models. All R2Y and Q2 values to the left were lower than the original points to the right of the blue regression line of the Q2 points, which intersects the vertical axis (on the left) at or below zero, showing that the PLS-DA model was valid. Permutation test plot for the PLS-DA model (number of permutations, 200; intercepts R2=0.0, 0.64, Q2=0.0, -0.159 in ESI+; R2=0.0, 0.829, Q2=0.0, -0.162 in ESI−). ESI, electrospray ionization; PLS-DA, partial least squares-discriminant analysis.

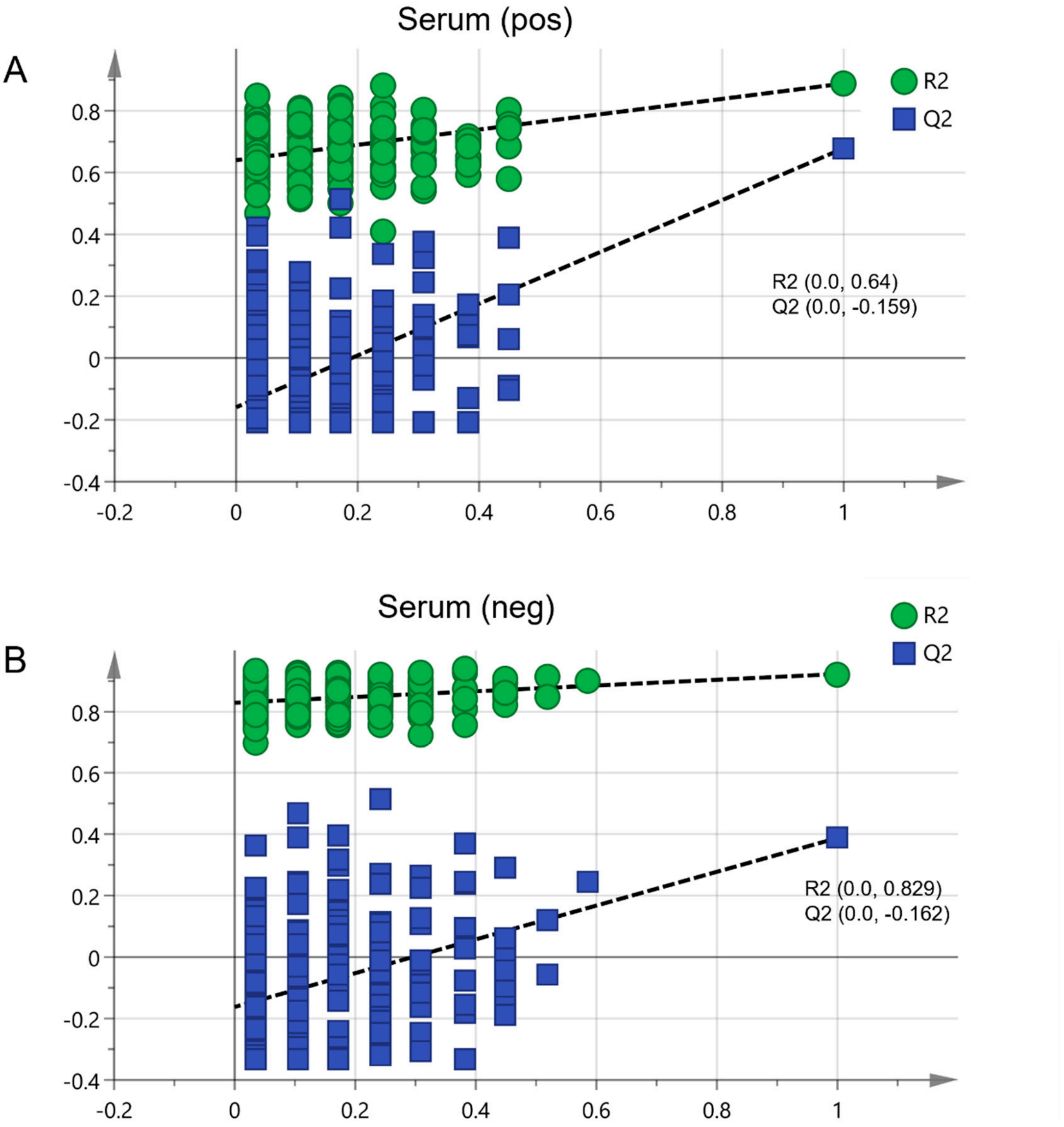

Supplement: Supplementary file 1 [file nutrients-15-02984-s001.zip › Supporting Information-submitted.pdf]
