# Supplementary material for: Alterations in Faecal and Serum Metabolic Profiles in Patients with Neovascular Age-Related Macular Degeneration
Source: Nutrients. 2023 Jun 30;15(13):2984. doi: 10.3390/nu15132984 (PMC10346323; doi:10.3390/nu15132984)
Supplement: Supplementary file 1 [file nutrients-15-02984-s001.zip › Table S1&S2.pptx]

## Slide 1
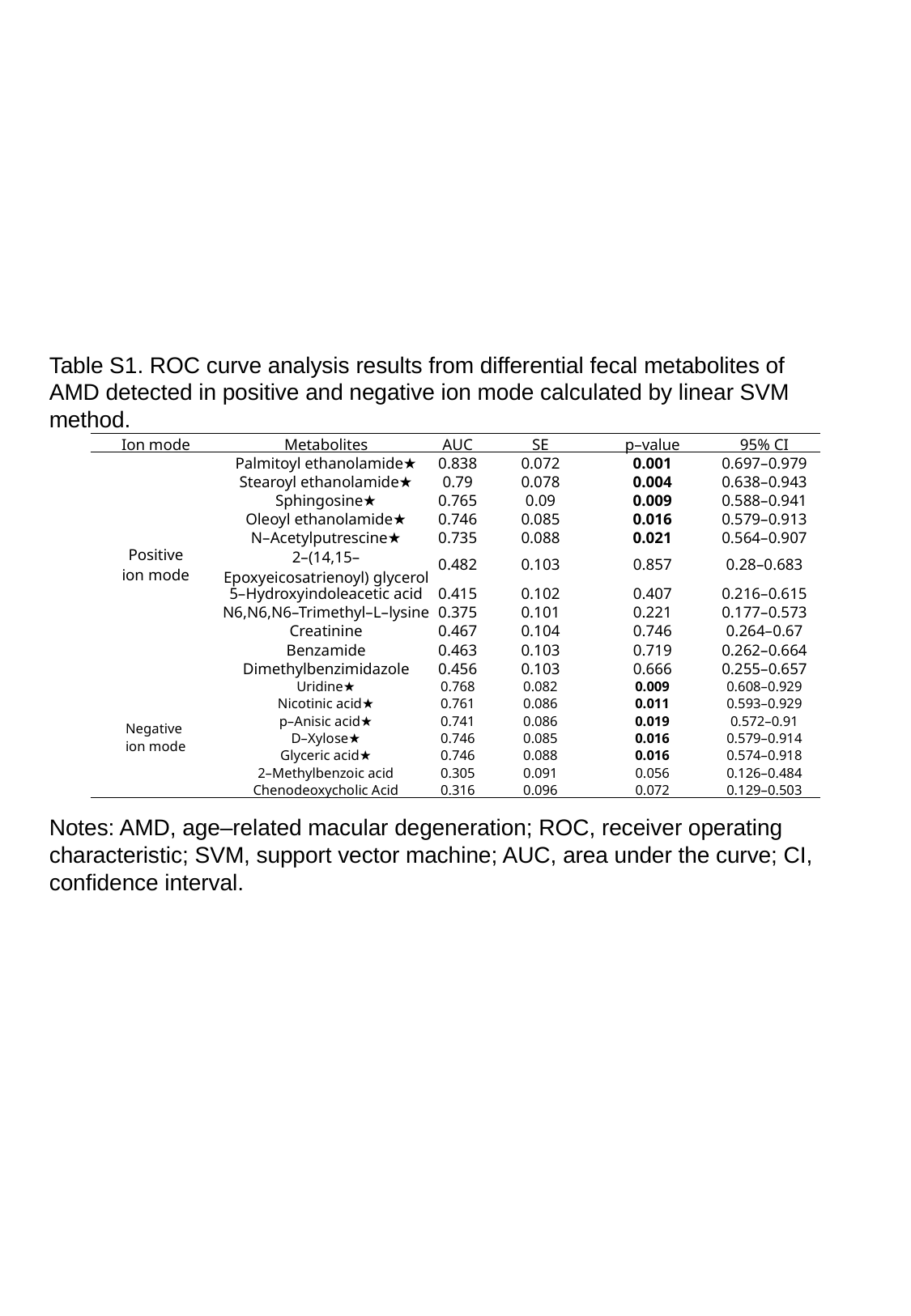

Table S1. ROC curve analysis results from differential fecal metabolites of AMD detected in positive and negative ion mode calculated by linear SVM method.
| Ion mode | Metabolites | AUC | SE | p–value | 95% CI |
| --- | --- | --- | --- | --- | --- |
| Positive ion mode | Palmitoyl ethanolamide★ | 0.838 | 0.072 | 0.001 | 0.697–0.979 |
| | Stearoyl ethanolamide★ | 0.79 | 0.078 | 0.004 | 0.638–0.943 |
| | Sphingosine★ | 0.765 | 0.09 | 0.009 | 0.588–0.941 |
| | Oleoyl ethanolamide★ | 0.746 | 0.085 | 0.016 | 0.579–0.913 |
| | N–Acetylputrescine★ | 0.735 | 0.088 | 0.021 | 0.564–0.907 |
| | 2–(14,15–Epoxyeicosatrienoyl) glycerol | 0.482 | 0.103 | 0.857 | 0.28–0.683 |
| | 5–Hydroxyindoleacetic acid | 0.415 | 0.102 | 0.407 | 0.216–0.615 |
| | N6,N6,N6–Trimethyl–L–lysine | 0.375 | 0.101 | 0.221 | 0.177–0.573 |
| | Creatinine | 0.467 | 0.104 | 0.746 | 0.264–0.67 |
| | Benzamide | 0.463 | 0.103 | 0.719 | 0.262–0.664 |
| | Dimethylbenzimidazole | 0.456 | 0.103 | 0.666 | 0.255–0.657 |
| Negative ion mode | Uridine★ | 0.768 | 0.082 | 0.009 | 0.608–0.929 |
| | Nicotinic acid★ | 0.761 | 0.086 | 0.011 | 0.593–0.929 |
| | p–Anisic acid★ | 0.741 | 0.086 | 0.019 | 0.572–0.91 |
| | D–Xylose★ | 0.746 | 0.085 | 0.016 | 0.579–0.914 |
| | Glyceric acid★ | 0.746 | 0.088 | 0.016 | 0.574–0.918 |
| | 2–Methylbenzoic acid | 0.305 | 0.091 | 0.056 | 0.126–0.484 |
| | Chenodeoxycholic Acid | 0.316 | 0.096 | 0.072 | 0.129–0.503 |
Notes: AMD, age–related macular degeneration; ROC, receiver operating characteristic; SVM, support vector machine; AUC, area under the curve; CI, confidence interval.

## Slide 2
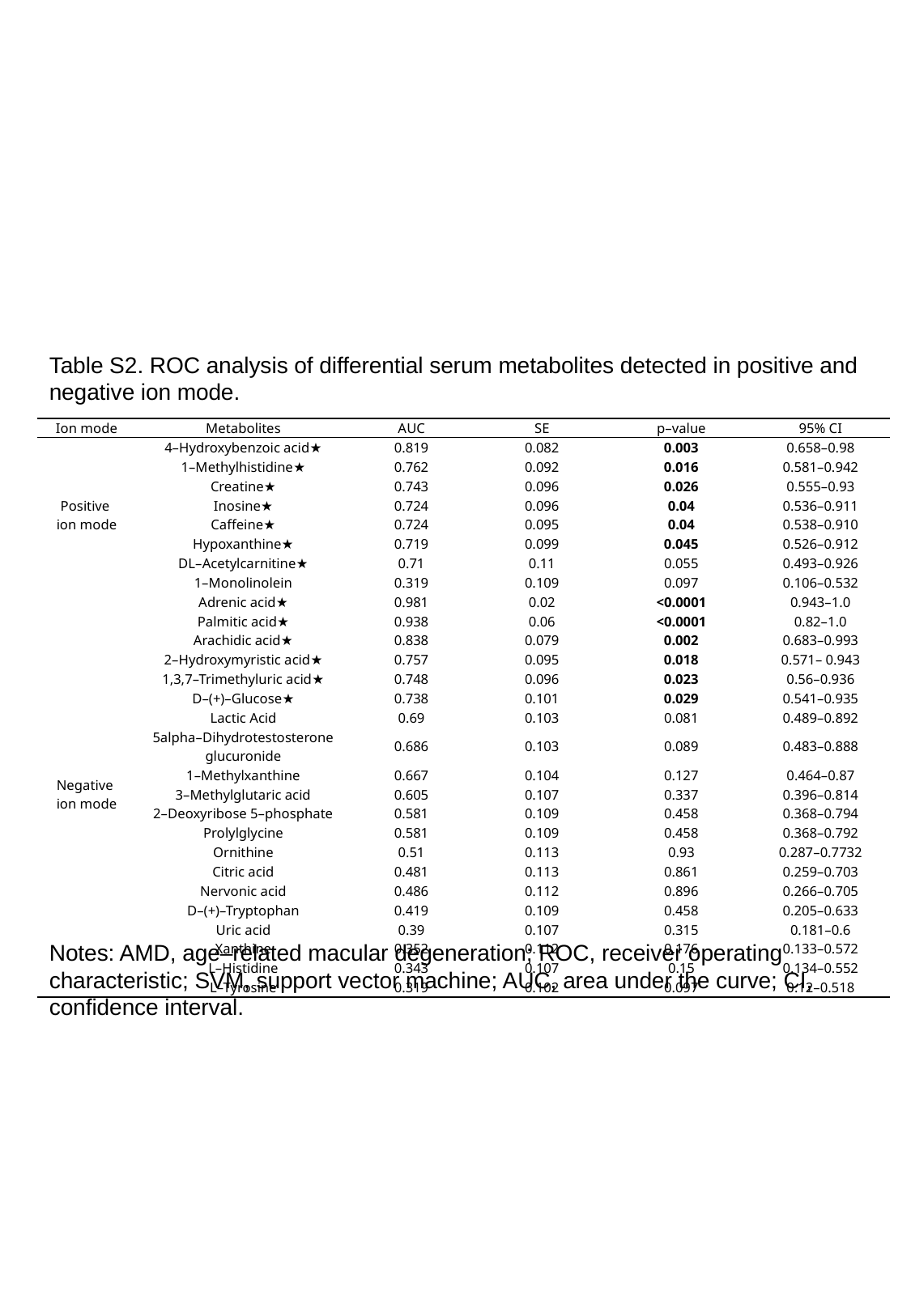

Table S2. ROC analysis of differential serum metabolites detected in positive and negative ion mode.
| Ion mode | Metabolites | AUC | SE | p–value | 95% CI |
| --- | --- | --- | --- | --- | --- |
| Positive ion mode | 4–Hydroxybenzoic acid★ | 0.819 | 0.082 | 0.003 | 0.658–0.98 |
| | 1–Methylhistidine★ | 0.762 | 0.092 | 0.016 | 0.581–0.942 |
| | Creatine★ | 0.743 | 0.096 | 0.026 | 0.555–0.93 |
| | Inosine★ | 0.724 | 0.096 | 0.04 | 0.536–0.911 |
| | Caffeine★ | 0.724 | 0.095 | 0.04 | 0.538–0.910 |
| | Hypoxanthine★ | 0.719 | 0.099 | 0.045 | 0.526–0.912 |
| | DL–Acetylcarnitine★ | 0.71 | 0.11 | 0.055 | 0.493–0.926 |
| | 1–Monolinolein | 0.319 | 0.109 | 0.097 | 0.106–0.532 |
| Negative ion mode | Adrenic acid★ | 0.981 | 0.02 | <0.0001 | 0.943–1.0 |
| | Palmitic acid★ | 0.938 | 0.06 | <0.0001 | 0.82–1.0 |
| | Arachidic acid★ | 0.838 | 0.079 | 0.002 | 0.683–0.993 |
| | 2–Hydroxymyristic acid★ | 0.757 | 0.095 | 0.018 | 0.571– 0.943 |
| | 1,3,7–Trimethyluric acid★ | 0.748 | 0.096 | 0.023 | 0.56–0.936 |
| | D–(+)–Glucose★ | 0.738 | 0.101 | 0.029 | 0.541–0.935 |
| | Lactic Acid | 0.69 | 0.103 | 0.081 | 0.489–0.892 |
| | 5alpha–Dihydrotestosterone glucuronide | 0.686 | 0.103 | 0.089 | 0.483–0.888 |
| | 1–Methylxanthine | 0.667 | 0.104 | 0.127 | 0.464–0.87 |
| | 3–Methylglutaric acid | 0.605 | 0.107 | 0.337 | 0.396–0.814 |
| | 2–Deoxyribose 5–phosphate | 0.581 | 0.109 | 0.458 | 0.368–0.794 |
| | Prolylglycine | 0.581 | 0.109 | 0.458 | 0.368–0.792 |
| | Ornithine | 0.51 | 0.113 | 0.93 | 0.287–0.7732 |
| | Citric acid | 0.481 | 0.113 | 0.861 | 0.259–0.703 |
| | Nervonic acid | 0.486 | 0.112 | 0.896 | 0.266–0.705 |
| | D–(+)–Tryptophan | 0.419 | 0.109 | 0.458 | 0.205–0.633 |
| | Uric acid | 0.39 | 0.107 | 0.315 | 0.181–0.6 |
| | Xanthine | 0.352 | 0.112 | 0.176 | 0.133–0.572 |
| | L–Histidine | 0.343 | 0.107 | 0.15 | 0.134–0.552 |
| | L–Tyrosine | 0.319 | 0.102 | 0.097 | 0.12–0.518 |
Notes: AMD, age–related macular degeneration; ROC, receiver operating characteristic; SVM, support vector machine; AUC, area under the curve; CI, confidence interval.
